# Disease Biomarkers in Gastrointestinal Malignancies

**DOI:** 10.1155/2016/4714910

**Published:** 2016-07-03

**Authors:** Omeed Moaven, Hamid Raziee, Wilbur Bowne, Mohammad Reza Abbaszadegan, Bryan C. Fuchs

**Affiliations:** ^1^Department of Surgery, University of Alabama, Birmingham, AL 35233, USA; ^2^Department of Radiation Oncology, University of Toronto, Toronto, ON, Canada M5G 2M9; ^3^Department of Surgery, Drexel University, Philadelphia, PA 19102, USA; ^4^Medical Genetics Research Center, Mashhad University of Medical Sciences, Mashhad 91967, Iran; ^5^Department of Surgery, Massachusetts General Hospital, Boston, MA 02114, USA

Of every five newly diagnosed cancers, one is a gastrointestinal (GI) malignancy in origin. Lower GI cancers are among the top three most frequent cancers in the United States and many western countries while upper GI cancers rank as the most prevalent type in many Asian countries, especially in central and eastern Asia. GI cancers are usually diagnosed in more advanced stages and in the absence of effective early diagnostic tools and therapeutic modalities, the survival rates are generally disappointingly low.

Considering the high mortality rate, tremendous effort has been directed to address the urgent need for discovery of effective early diagnostic tools, efficient therapeutic targets, and treatment monitoring markers for GI malignancies. Biomarkers are one of these favorite tools with several potential applications in various aspects of clinical management of cancers. A plethora of biomarkers has been studied in GI cancers, of which only a handful have found their way from bench to bed. Guidelines have been published by different cancer societies and groups, such as American Society of Clinical Oncology (ASCO), European Society of Medical Oncology (ESMO), and European Group on Tumour Markers (EGTM), with recommendations regarding clinical applications of available markers for gastrointestinal tumors [1–3]. CEA,* K-RAS*, HER2, and KIT are among the biomarkers with validated clinical implications in management of colorectal cancer (CRC), gastric cancer, and gastrointestinal stromal tumors [1, 4–6]. Nonetheless, there is a growing list of emerging markers with promising clinical results that need to be validated for routine clinical applications and current data are insufficient to recommend them as part of the clinical guidelines.

The current special issue tackles this important area of cancer research. In this issue, S.-F. Chiang et al. report their investigation of bone marrow stromal antigen 2 (BST2; also known as CD317, tetherin, and HM1.24) as a plasma biomarker in 152 patients with CRC. They show that, compared to the controls, BST2 was significantly elevated in plasma samples from CRC patients. In addition, high BST2 expression in CRC tissue, as assessed by immunohistochemistry, was associated with poorer 5-year survival. BST2 has also been under investigation as a potential target for immunotherapy for over a decade [7]. In fact, a humanized monoclonal antibody targeting BST2 has been tested in Phase 1 trial of multiple myeloma (MM) but the response rate was low [8]. More recently, BST2-specific cytotoxic T lymphocytes targeting MM cells have been developed [9, 10]. Therefore, it is possible that BST2 could be a potential therapeutic target in CRC. However, given its detection in the plasma, future studies should also examine BST2 as a novel biomarker to noninvasively monitor therapeutic response.

In another study, T. Xue et al. have investigated the clinical significance of miRNA-20b as a marker in hepatocellular carcinoma (HCC) and reported its association with poor survival. They confirmed HIF-1*α* and VEGF as the targets of miRNA-20b* in vitro* and showed their regulation in both normal and hypoxic conditions, suggesting miRNA-20b as an adaptation mechanism that may play a role in tumor progression. This study was performed on a small retrospective cohort and the intriguing results should be validated in future larger prospective studies. Also the functional studies need to be expanded to better understand its role in tumor progression.

Thomsen-Friedenreich (TF) antigen is one of the tumor-associated glycans (TAG), which is normally overexpressed in cancer cells, and has a role in cell adhesion to endothelium. In search of a serologic biomarker for gastric cancer, O. Kurtenkov and K. Klaamas looked into the presence and avidity of anti-TF antibodies in serum samples of cancer patients and normal controls. Drawing on their prior study showing increased sialylation of anti-TF antibodies in gastric cancer, they assessed the following: (1) serum levels of anti-TF antibodies by ELISA; (2) reactivity of anti-TF antibodies to* Sambucus nigra* agglutinin (SNA); (3) avidity of anti-TF antibodies by ELISA; and (4) avidity of SNA-reactive anti-TF antibodies in 104 patients and 49 controls. They showed, for the first time, that SNA-reactive—and therefore aberrantly sialylated—TF-specific antibodies have a significantly higher avidity in cancer patients, with a diagnostic accuracy of 73.2%, and a sensitivity of 70.3% in stage I patients. While these results provide an exciting venue of further investigation for a serum-based marker for gastric cancer, all these biomarkers need prospective evaluation and validation studies for determining the clinical impact, which is missing for many of the newly diagnosed markers. The clinical application would need stringent prospective validation of specificity, sensitivity, and cost-effectiveness.

The neutrophil-to-lymphocyte ratio (NLR) has been proposed as a potential inflammation-based prognostic indicator in various malignancies but there have been controversial reports of its prognostic values in gastric cancer. Sun et al. are here reporting the results of a meta-analysis including 19 studies with 5431 patients and concluded that pretreatment NLRs can predict the prognosis of gastric cancer. The clinical significance of these findings still needs to be validated in a larger independent study.

In an effort to highlight the implications of HER2, a marker which is now accepted as part of practice guidelines in advanced gastric cancer, A. Ieni et al. have reviewed the HER2 status in various stages of gastric tumorigenesis and their clinical significance, suggesting a potential role in early steps of gastric carcinogenesis and offering potential clinical implications in both early and advanced gastric adenocarcinoma.

Further on serum-based markers H. Kishikawa et al. review the current evidence about the use of “ABC method,” a combination of anti-*Helicobacter pylori* antibody and serum pepsinogen (PG), for gastric cancer screening. In this method, based on* H. pylori* (HP) titre and PG, subjects are subdivided into 4 groups (A, HP−/PG−; B, HP+/PG−; C, HP+/PG+; D, HP−, PG+), with recommendation for endoscopy surveillance in B, C, and D groups every 3, 2, and 1 year, respectively. After discussing the available evidence, the authors conclude that gastric cancer risk is not the same in each of the above categories and recommend that HP antibody titre measurement should be done to categorize patients into low-negative, high-negative, low-positive, and high-positive groups. They further recommend endoscopic surveillance in high-negative antibody titres in group A every 3 years, high-positive titres in group B every 2 years, and low-positive titres in group C every year.

Recommending a tumor marker as part of a practice guideline requires a multistep complex process that starts with discovery and introduction of the biomarker in preclinical phase followed by a rigorous analytical validation that comprises assay development, strong methodology, and robust statistical and bioinformatics tools. The ultimate path toward FDA or other regulatory approval is an unequivocal clinical validation with independent prospective studies. This process can take two to three decades and there are many examples of overoptimistic interpretation of the promising early results [11–13], which eventually failed to succeed achieving FDA clearance due to lack of accuracy or robustness in at least one of the above-mentioned steps. While we all review, observe, and contribute to the expanding body of literature of the emerging tumor markers, learning the lessons from the stories of failures and successes will create a pragmatic and realistic path toward the ultimate goal of recognizing a tumor marker as an effective tool with a significant clinical outcome.

*Omeed Moaven* *Omeed Moaven* *Hamid Raziee* *Hamid Raziee* *Wilbur Bowne* *Wilbur Bowne* *Mohammad Reza Abbaszadegan* *Mohammad Reza Abbaszadegan* *Bryan C. Fuchs* *Bryan C. Fuchs*
